# Effect of Combination of Traditional Chinese Medicine with Western Medicine on Endometrial Carcinoma and Its Influence on Ultrasound, MRI, Tumor Markers HE4 and CA125

**DOI:** 10.1155/2021/6053406

**Published:** 2021-12-02

**Authors:** Xia Gao, Qiuying Li, Yanwen Qu, Jinzhi Zhang, Yougang Xing, Shichun Li

**Affiliations:** ^1^Department of Nursing, Zhangqiu Maternity and Child Care Hospital, Jinan 250200, China; ^2^Department of Imaging, Zhangqiu District People's Hospital, Jinan 250200, China; ^3^Department of Female Tumor, Affiliated Qingdao Central Hospital, Qingdao University, Qingdao 266000, China; ^4^Department of Ultrasound, Zhangqiu District People's Hospital, Jinan 250200, China

## Abstract

**Objective:**

To study the clinical efficacy of integrated traditional Chinese medicine (TCM) and Western medicine (WM) in treating endometrial cancer and the influence on ultrasound, magnetic resonance imaging (MRI), tumor markers, human epididymis protein 4 (HE4) and carbohydrate antigen 125 (CA125).

**Method:**

A total of 152 cases of patients with endometrial carcinoma were randomly divided into two groups: the TCM + WM group and the WM group. The WM group was treated with megestrol acetate tablets, and the TCM + WM group was treated with Radix Astragali injection on the basis of the control group. The levels of inflammatory factors, HE4 and CA125 in serum, were detected using enzyme-linked immunosorbent assay (ELISA) or radioimmunoassay. The characteristics of ultrasound images and MRI images were observed and recorded. Toxicity, side effects, and the 3-year cumulative survival rate after treatment were assessed.

**Results:**

After treatment, the levels of interleukin-4 (IL-4), tumor necrosis factor-alpha (TNF-*α*), and high-sensitivity C-reactive protein (hs-CRP) in both groups decreased, and the decrease in the TCM + WM group was more obvious than that in the WM group. There were statistically significant differences between the two groups in lesion shape, boundary, blood flow signal, lesion diameter, resistance index (RI), echo, intima thickness, and muscle layer infiltration from transvaginal ultrasound images after treatment. The diameter, echo, boundary, shape, composition, and enhancement degree of lesions between the two groups have a significant difference. Moreover, the levels of serum HE4 and CA125 in both groups decreased after treatment, and the decrease in the TCM + WM group was more obvious than that in the WM group. There were statistically significant differences between the two groups in the occurrence of myelosuppression, abnormal liver function, decreased platelet number, gastrointestinal reactions, leukopenia, and cardiotoxicity. After three years of follow-up, the cumulative survival rate of the TCM + WM group was 76.32%, and the cumulative survival rate of the WM group was 57.89%.

**Conclusion:**

Radix Astragali injection combined with megestrol acetate tablets has obvious therapeutic effects against endometrial cancer. Through vaginal ultrasonography and MRI, it can significantly improve the size, shape, and blood flow signals of patients' lesions, reduce the level of serum inflammatory factors and tumor markers HE4 and CA125, reduce the incidence of toxic and side reactions, improve the patient's immunity, improve the patient's condition significantly, and prolong the survival time of patients.

## 1. Introduction

Endometrial cancer is a malignant tumor of the female reproductive system, which occurs more frequently during the perimenopausal and postmenopausal periods of women, and the main symptoms are vaginal bleeding and menstrual disorders [1]. At present, the disease has a high morbidity and mortality rate and is showing a continuous growth trend worldwide, and the incidence is increasing at a young age, which seriously threatens the physical and mental health of most female patients [2]. Radiotherapy and chemotherapy are currently important adjuvant treatments for endometrial cancer, which mainly consist of cytotoxic drugs to directly target the growth and reproduction of cancer cells, thereby inhibiting and killing cancer cells [3]. Megestrol acetate can act on proliferative cancer cells, binding estrogen receptors, blocking their binding with estrogen, and inhibiting endometrial cancer cell proliferation and metastasis [4]. Although this treatment method can eliminate cancer cells in the patient's body, it has a cytotoxic effect on normal cells in the proliferation stage, causing adverse clinical symptoms such as leukopenia and myelosuppression in the patient's body, reducing the patient's tolerance to treatment, and having high toxic side effects [5]. TCM has certain advantages in enhancing efficacy and reducing the toxic effects of chemotherapy and has been widely used in the adjuvant treatment of chemotherapy for endometrial cancer in recent years [6, 7]. Studies have shown that the combination of TCM injection and chemotherapy can reduce the side effects of chemotherapy, and patients who use Western medicine for antitumor therapy can still use TCM injection to stabilize and strengthen the therapeutic effects [8]. Radix Astragali injection is one of the TCM injections in the National Medical Insurance Catalog. It can be combined with chemotherapy to improve the patients' immunity and improve the quality of life of patients [9]. In recent years, as an immune enhancer, Radix Astragali injection has been used in the clinical treatment of various diseases (such as ovarian and breast cancer) and has achieved good therapeutic outcomes and antitumor effects [10, 11]. However, its therapeutic outcomes in endometrial cancer are rarely reported. Studies have found that serum levels of CA125 and HE4 are closely related to the onset and development of endometrial cancer [12, 13]. This study explores the efficacy of integrated TCM and WM in endometrial cancer.

## 2. Materials and Methods

### 2.1. The General Information

A total of 152 endometrial cancer patients admitted to Zhangqiu Maternity and Child Care Hospital, Jinan, Shandong, China, from December 2015 to January 2018 were selected, all of whom were diagnosed by pathological biopsy. The inclusion criteria were as follows: (1) meet the diagnostic criteria for endometrial cancer [14]; (2) clinical survival >3 months; (3) the lesion tissue can be clinically detected, with symptoms such as irregular vaginal bleeding and lower abdominal pain; and (4) no symptoms of allergy to the study drug. The exclusion criteria were as follows: (1) the presence of mental and cognitive disorders; (2) complicated with serious dysfunction of the heart, lung, and kidney; (3) patients who received radiotherapy, chemotherapy, or traditional Chinese medicine 3 months before enrollment; (4) complicated with other malignant tumors, accompanied by breast lumps and other adverse symptoms; and (5) uterine fibroids and fallopian tube cancer during lactation or pregnancy. All patients were randomly divided into the Western medicine (WM) group and the integrated traditional Chinese and Western medicine (TCM + WM) group, with 76 cases in each group. The age of the WM group was 37–68 years old, with an average of 51.4 ± 8.1 years old; the course of disease was 3–12 months, with an average of 8.22 ± 2.74 months; and cancer types were as follows: 8 cases of clear cell carcinoma, 26 cases of adenosquamous carcinoma, and 42 cases of adenocarcinoma. The age of the TCM + WM group was 38– 70 years, with an average of 49.6 ± 8.6 years; the course of disease was 4–13 months, with an average of (8.48 ± 2.84) months; cancer types were as follows: 10 cases of clear cell carcinoma, 27 cases of adenosquamous carcinoma, and 39 cases of glandular cancer. There was no statistically significant difference between the two groups' general information (*P* > 0.05), and they were comparable. This study was reviewed and approved by the Medical Ethics Committee of Zhangqiu Maternity and Child Care Hospital, and the patients and their families had informed consent and signed an informed consent form. This study was conducted in accordance with the Declaration of Helsinki.

### 2.2. Treatment Method

After admission, the two groups of patients underwent routine examinations under the supervision of medical staff and were treated according to their actual conditions. Both groups were given 80 mg paclitaxel (Haikou Qili Pharmaceutical Co., Ltd., drug approval number: H20063169) and 40 mg cisplatin (Qilu Pharmaceutical Co., Ltd., drug approval number: H37021358) for basic treatment, a total of 4 chemotherapy treatments. At the same time, patients in the WM group were given oral megestrol acetate tablets (Shanghai Xinyi Tianping Pharmaceutical Co., Ltd., drug approval number: H20053712) for treatment, 2 times/d, 80 mg/time for 12 weeks. On the basis of the WM group, the patients of the TCM + WM group were treated with Huangqi injection (Drug approval number, Heilongjiang Zhenbaodao Pharmaceutical Co., Ltd., 2 ml/branch), intramuscular injection, 3 ml/time, 1 time/d, 21 days as a cycle, 3 consecutive chemotherapy cycles.

### 2.3. Ultrasonography

All patients were examined by using GE E8 color Doppler ultrasonography with a probe frequency of 3.5∼8 MHz. The bladder was kept full before scanning, and the bladder lithotomy position was taken. Transvaginal ultrasonography was performed routinely to carefully observe the endometrial morphology, intrauterine lesion morphology, size, internal echo, and muscle layer infiltration depth. Color Doppler flow imaging (CDFI) mode was used to observe the blood flow of the lesion in multiple sections and measure the blood RI value.

### 2.4. MRI Examination

All patients were examined using a GE 1.5T superconducting MRI scanner with an 8-channel phased array coil. Before the examination, the patient kept his bladder properly filled and laid in a supine position with a routine plain scan. The scan sequences included transection, coronal planes, sagittal plane autogyro wave T2W1 and T2W1 fat suppression sequence, and transection autogyro wave T1W1 sequence. After plain scanning, enhanced scanning was performed. The contrast agent gadolinium spray meglumine (0.1 mmol/kg) was injected intravenously to analyze tumor morphology, size, echo, and signal intensity under different sequence scanning.

### 2.5. Observation Index

(1) The serum inflammatory factors and tumor marker levels were compared between the two groups before and after treatment. 5 ml of fasting venous blood of the two groups in the morning was collected before and after treatment, and centrifuged in a test tube for 5 min to obtain the supernatant. The contents of TNF-*α*, hs-CRP, IL-4, and HE4 in serum were determined by enzyme-linked immunosorbent assay (ELISA), and the content of tumor marker CA125 in serum was determined by radioimmunoassay. Reagents were provided by Roche Reagent Co., Ltd. of Germany. (2) Transvaginal color ultrasound image features and MRI image features of patients in the two groups were observed. The shape, diameter, boundary, echo, blood flow signal, and enhancement degree of the lesion were recorded. (3) The incidence of toxic and side effects was compared between the two groups, including myelosuppression, abnormal liver and kidney function, decreased platelet number, gastrointestinal reactions, leukopenia, and cardiotoxicity. (4) Three-year cumulative survival rates were compared between the two groups. Through monthly telephone follow-up, the patient's condition and survival were inquired and recorded, and the three-year cumulative survival rate was calculated.

### 2.6. Statistical Analysis

SPSS 23.0 software was used for data analysis. Counting data were expressed as examples or percentages, measurement data were expressed as $\bar{x}\pm s$, and *χ*^2^ test was used. *P* < 0.05 means the difference is statistically significant.

## 3. Results

### 3.1. Comparison of the Levels of Inflammatory Factors before and after Treatment between the Two Groups

**Figure 1:**
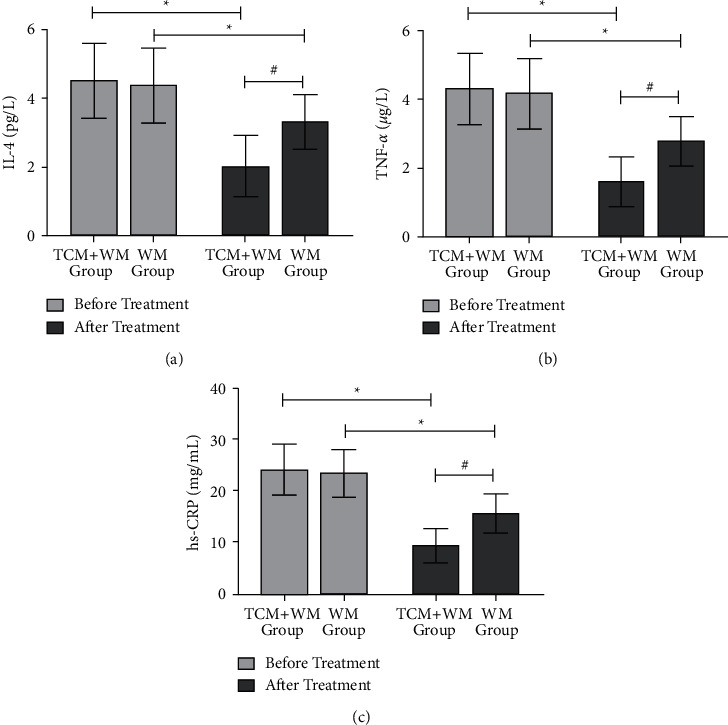
Comparison of inflammatory factors levels between the two groups before and after treatment (^*∗*^compare with before treatment, *P* < 0.05; ^#^compare with the control group, *P* < 0.05). (a) The comparison of IL-4 levels between the two groups before and after treatment. (b) The comparison of TNF-*α* levels between the two groups before and after treatment. (c) The comparison of hs-CRP levels between the two groups before and after treatment.

There were no significant differences in serum IL-4, TNF-*α*, and hs-CRP levels between the two groups before treatment (Figure 1). After treatment, the levels of serum IL-4, TNF-*α*, and hs-CRP in two groups decreased compared with before treatment, and the decrease in the TCM + WM group was more obvious than in the WM group (Figure 1).

### 3.2. Comparison of the Characteristics of Transvaginal Color Doppler Ultrasound Images before and after Treatment between the Two Groups

Before treatment, there was no statistically significant difference in the characteristics of transvaginal color Doppler ultrasound images between the two groups (*P* > 0.05) (Table 1). After treatment, the two groups of patients had statistically significant differences in lesion shape, boundary, blood flow signal, lesion diameter, RI, echo, intimal thickness, and muscle infiltration (*P* < 0.05) (Table 2).

### 3.3. Comparison of MRI Image Characteristics between the Two Groups before and after Treatment

Before treatment, there was no statistically significant difference in MRI features between the two groups (*P* > 0.05) (Tables 3). After treatment, there were statistically significant differences in lesion diameter, echo, boundary, shape, composition, and enhancement degree between the two groups (*P* < 0.05) (Table 4).

### 3.4. Comparison of Serum CA125 and HE4 Expression Levels between the Two Groups before and after Treatment

**Figure 2:**
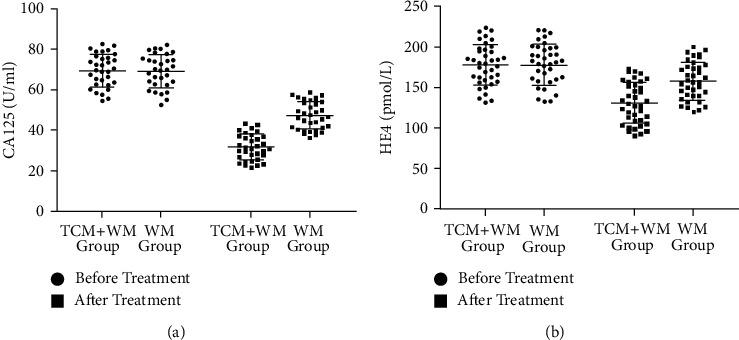
Comparison of serum (CA125 and HE4) expression levels between the two groups before and after treatment. (a) The comparison of serum CA125 expression levels between the two groups before and after treatment. (b) The comparison of HE4 expression levels between the two groups before and after treatment.

There was no significant difference in serum (CA125 and HE4) expression levels between the two groups before treatment (*P* > 0.05) (Figure 2). After treatment, the expression levels of serum CA125 and HE4 in both groups decreased compared with before treatment (*P* < 0.01), and the decrease of the expression levels of serum in the TCM + WM group was more obvious than those in the WM group; the difference was statistically significant (*P* < 0.01) (Figure 2).

### 3.5. Comparison of Toxicities and Side Effects between the Two Groups

After treatment, there were 2 cases of myelosuppression, 3 cases of abnormal liver function, 2 cases of decreased platelet number, 1 case of gastrointestinal reaction, 2 cases of leukopenia, and 3 cases of cardiotoxicity in the TCM + WM group (Table 5). In the WM group, there were 7 cases of myelosuppression, 9 cases of abnormal liver function, 9 cases of decreased platelet number, 10 cases of gastrointestinal reaction, 11 cases of leukopenia, and 8 cases of cardiotoxicity (Table 5). The difference was statistically significant (*P* < 0.05) (Table 5).

### 3.6. Comparison of 3-Year Cumulative Survival Rates between the Two Groups

**Figure 3:**
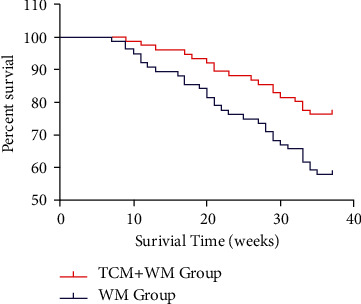
Comparison of 3-year cumulative survival rates between the two groups.

After three years of follow-up, 18 patients in the TCM + WM group died, and the cumulative survival rate was 76.32%, while 32 patients in the WM group died, and the cumulative survival rate was 57.89%. The difference was statistically significant (*P* < 0.05) (Figure 3).

## 4. Discussion

Endometrial cancer is a malignant tumor disease that occurs in the epithelium of the endometrium. Its main clinical manifestations include vaginal bleeding, vaginal discharge, and lower abdominal pain, and lymph node metastasis and distant organ metastasis can also occur in the late stage of the disease [1]. Endometrial cancer has many pathogenic factors, and its incidence is closely related to lifestyle, but its specific etiology is still unclear. At present, surgical treatment is the primary treatment for endometrial cancer, but some patients who have surgical contraindications or refuse surgery for other reasons need to be treated with drugs to control the progression of the disease. Thus, finding more effective therapies for endometrial cancer has great significance.

At present, progesterone drugs are frequently used in the treatment of endometrial cancer. Megestrol is one of the commonly used drugs; it can effectively regulate hormones secreted by pituitary stimulation, inhibit the action of the female hormone secretion, at the same time also can block the body's hormone receptors from binding to estrogen, thus inhibiting the proliferation and diffusion of tumor cells, but also can promote the body cell differentiation, improve the clinical symptoms and prolong survival time [15, 16]. However, this treatment method has serious side effects and drug resistance problems [17]. As the quintessence of Chinese medicine, TCM plays a vital role in treating various diseases and has certain advantages in enhancing the efficacy and reducing the toxicity of chemotherapy. The combination of TCM and WM can reduce the side effects of radiotherapy and chemotherapy and improve patients' quality of life. According to TCM approaches, endometrial cancer is a systemic disease although its lesion site is in the uterus, which is a syndrome of asthenia in origin and excess in superficiality caused by the dysfunction of zang-fu organs and poor blood flow [9]. The main purpose of treatment is to promote blood circulation, remove blood stasis, clear heat, and detoxify. Radix Astragali injection is an effective extract of Astragalus. Modern pharmacological investigations have confirmed that it has immunomodulatory, anti-infective, antioxidant, antiviral, antitumor, and hypoglycemic effects and can regulate blood pressure bidirectionally and protect various organs such as the lung, spleen, liver, and kidney [9].

IL-4 is mainly produced by activated T cells. It can not only participate in inflammatory reactions but also increase the number of platelets, make the patient's blood coagulation function abnormal, cause blood flow to slow down, increase the accumulation of tumor cells in circulation, and cause the damage of the basement membrane to transfer to other tissues and organs [18]. TNF-*α* is produced by macrophages and can act on vascular endothelial cells, causing damage to endothelial cells and vascular inflammation, resulting in changes in vascular function and the development and metastasis of tumor cells [19]. Hs-CRP, an inflammatory response protein produced by the liver, is a marker of inflammation in the acute stage and can cause vascular endothelial damage and tumor angiogenesis [20]. The results of this study showed that the levels of IL-4, TNF-*α*, and Hs-CRP in both groups decreased after treatment, and the decrease was more evident in the TCM + WM group than in the WM group. These results indicated that the combination of TCM and WM in the treatment of endometrial cancer could inhibit the release of inflammatory factors, significantly improve the inflammatory environment in vivo, and thus inhibit tumor cell growth, which plays an essential role in adjuvant therapy.

CA125 is an important tumor marker, widely used in the early diagnosis and clinical prognosis evaluation of malignant tumors [21–23]. Studies have reported that the high level of CA125 is related to the occurrence and development of endometrial cancer [24]. HE4, as a newly discovered tumor marker for endometrial cancer, is a secretory glycoprotein with high sensitivity and specificity [25]. Studies have documented that serum HE4 and CA125 levels can reliably predict the poor prognosis of patients with endometrial cancer [26]. Li et al. pointed out that serum HE4 levels can be used as an indicator for early diagnosis of endometrial cancer [27]. Other studies have shown that the combined detection of HE4 and CA125 can effectively improve the diagnostic accuracy and sensitivity of endometrial cancer [12]. Here, integrated TCM and WM can significantly reduce serum HE4 and CA125 levels, thus decreasing tumor activity and inhibiting tumor proliferation and growth in patients with endometrial cancer. It is further indicated that the combination of TCM and WM in the treatment of endometrial cancer can reduce the toxicities and side effects, balance the platelets and white blood cell numbers in the patient, and protect the bone marrow at the same time, which is of high safety, and can also induce the cancer cell apoptosis and prolong the life cycle. Transvaginal ultrasound can not only display two-dimensional ultrasound images but also further observe the nature of blood flow, speed, and other information, so as to sensitively and accurately reflect the pathological changes of the endometrium [28, 29]. MRI has high tissue resolution and can accurately assess the degree and extent of lesion invasion in patients with endometrial cancer [30, 31]. The two detection methods are not invasive and are more acceptable to patients. The results of the two detection methods can further confirm that the treatment of endometrial cancer with integrated TCM and WM has good clinical efficacy, effectively clearing the focus and improving patients' clinical symptoms and immunity.

## 5. Conclusion

Radix Astragali injection combined with megestrol treatment of endometrial cancer has certain clinical curative effects. It can enhance immunity, effectively control the development condition, improve the quality of life, reduce the inflammatory response and CA125 and HE4 levels, inhibit tumor cell growth, reduce the size of the lesion area, and prolong survival time.

## Figures and Tables

**Table 1:** Comparison of the characteristics of transvaginal ultrasound images between the two groups of patients before treatment (*n*).

Image characteristics	TCM + WM (*n* = 76)	WM (*n* = 76)	*χ* ^2^	*P*
Shape			0.110	0.740
Irregularity	47	45		
Agglomerate	29	31		

Boundary			0.365	0.546
Sharpness	14	17		
Obscure	62	59		

Blood flow signal			0.380	0.827
No	4	5		
Abundant	51	53		
Not abundant	21	18		

Lesion diameter			0.292	0.589
≥2 cm	53	56		
<2 cm	23	20		

RI			0.468	0.494
≤0.4	48	52		
>0.4	28	24		

Echo			0.249	0.618
Low	45	48		
Uneven	31	28		

Intimal thickness			0.128	0.721
≥1.5 cm	55	53		
<1.5 cm	21	23		

Muscle infiltration			0.272	0.873
None	5	6		
Shallow	32	34		
Deep	39	36		

**Table 2:** Comparison of characteristics of transvaginal ultrasound images between the two groups after treatment (*n*).

Image characteristics	TCM + WM (*n* = 76)	WM (*n* = 76)	*χ* ^2^	*P*
Shape			10.125	0.006
No	32	14		
Irregularity	27	39		
Agglomerate	17	23		

Boundary			6.779	0.009
Sharpness	49	33		
Obscure	27	43		

Blood flow signal			6.657	0.036
No	37	22		
Abundant	28	42		
Not abundant	11	12		

Lesion diameter			12.440	0.002
No	32	14		
≥2 cm	28	48		
<2 cm	16	14		

RI			13.633	<0.001
≤0.4	12	32		
>0.4	64	44		

Echo			16.067	<0.001
Low	16	39		
Uneven	26	20		
High	34	17		

Intimal thickness			6.410	0.011
≥1.5 cm	20	35		
<1.5 cm	56	41		

Muscle infiltration			7.916	0.019
None	39	22		
Shallow	18	26		
Deep	19	28		

**Table 3:** Comparison of MRI image features between the two groups before treatment (*n*).

Image characteristics	TCM + WM (*n* = 76)	WM (*n* = 76)	*χ* ^2^	*P*
Lesion diameter			0.032	0.857
≥2 cm	55	54		
<2 cm	21	22		

Echo			0.256	0.613
Low	47	50		
Uneven	29	26		

Boundary			0.158	0.691
Sharpness	17	15		
Obscure	59	61		

Shape			0.452	0.501
Irregularity	50	46		
Agglomerate	26	30		

Composition			0.106	0.744
Solid	35	33		
Cystic	41	43		

Enhancement degree			0.778	0.678
No	2	3		
Slightly	18	14		
Obviously	56	59		

**Table 4:** Comparison of MRI image features between the two groups after treatment (*n*).

Image characteristics	TCM + WM (*n* = 76)	WM (*n* = 76)	*χ* ^2^	*P*
Lesion diameter			6.147	0.046
No	30	16		
≥2 cm	33	42		
<2 cm	13	18		

Echo			6.531	0.038
Low	27	36		
Uneven	15	21		
High	34	19		

Boundary			8.622	0.003
Sharpness	51	33		
Obscure	25	43		

Shape			8.927	0.012
No	30	16		
Irregularity	24	41		
Agglomerate	22	19		

Composition			7.097	0.029
No	30	16		
Solid	17	28		
Cystic	29	32		

Enhancement degree			7.647	0.022
No	33	20		
Slightly	12	8		
Obviously	31	48		

**Table 5:** Comparison of the occurrence of toxicities and side effects between the two groups.

Group	Myelosuppression	Abnormal renal and liver function	Decreased platelet number	Gastrointestinal reactions	Leukopenia	Cardiotoxicity
TCM + WM	2 (2.63)	3 (3.95)	2 (2.63)	1 (1.32)	2 (2.63)	3 (3.95)
WM	7 (9.21)	9 (11.84)	9 (11.84)	10 (13.16)	11 (14.47)	8 (10.53)

## Data Availability

The datasets used and/or analyzed during the present study are available from the corresponding author on reasonable request.
